# Near-Complete Transection of the Sciatic Nerve After Closed Reduction Attempt of a Dislocated Total Hip Arthroplasty

**DOI:** 10.7759/cureus.51131

**Published:** 2023-12-26

**Authors:** Jacob Shermetaro, Ricardo Hernandez, Josiah Valk, Daniel McCall, Christopher Lumley

**Affiliations:** 1 Orthopedic Surgery, Corewell Health Farmington Hills, Farmington Hills, USA; 2 Plastic Surgery, Corewell Health Farmington Hills, Farmington Hills, USA

**Keywords:** hip closed reduction complications, total hip instability, sciatic nerve palsy, total hip closed reduction, dislocated total hip, sciatic nerve injury, case report

## Abstract

Sciatic nerve injuries are rare and devastating complications that can occur following total hip dislocations. These injuries are even more uncommon when resulting from a closed reduction attempt. In the literature, only one other case of sciatic nerve palsy secondary to sciatic nerve laceration has been reported. Conducting a careful neurovascular examination following a closed reduction procedure is crucial in determining the presence of sciatic nerve injury. We present a case of sciatic nerve palsy following a closed reduction attempt of a dislocated total hip arthroplasty (THA). Surgical exploration revealed a near-complete sciatic nerve laceration. The patient subsequently underwent neurolysis and nerve repair. This case highlights the importance of thorough neuromuscular examination following closed reduction of THA, with consideration for surgical exploration when necessary.

## Introduction

The sciatic nerve originates from the spinal nerves of L4 through S3 and consists of tibial and peroneal divisions, forming the largest nerve in the human body. It provides motor innervation to the posterior thigh and the entire lower leg. It is also responsible for the majority of the sensation over the lower leg except for a medial portion, which is innervated by the saphenous nerve [1]. Sciatic nerve injury is a known possible complication following native and prosthetic hip dislocations. However, these events are rare. The rate of dislocation after primary total hip arthroplasty (THA) varies from 1% to 3.2% and can reach as high as 7% or more following revision THA [2-4]. Sciatic nerve injuries following prosthetic hip dislocations have been reported to occur in about 0.1-3% of these cases [2,5,6]. Standard management of prosthetic hip dislocations includes closed reduction, given the low complication rate [7]. Although the literature is sparse on sciatic nerve recovery following these injuries, the prognosis is typically guarded.

We present a rare case of near-complete sciatic nerve transection following closed reduction attempts of a THA dislocation. To our knowledge, there are fewer than 10 documented cases in the literature of sciatic nerve palsy following closed reduction of THA dislocation. Only one of these case reports describes a lacerated sciatic nerve.

## Case presentation

**Figure 1:**
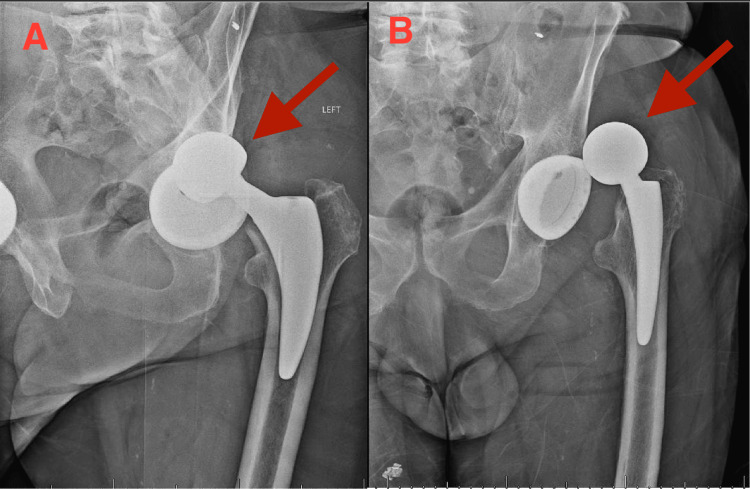
AP left hip radiograph (A) and oblique left hip radiograph (B) demonstrating anterior dislocation of left THA AP, anterior-posterior; THA, total hip arthroplasty

Informed consent was obtained from the patient for the use of this information for submission and publication. The patient is a 77-year-old male who underwent uncomplicated primary Mako-assisted left THA by a community surgeon. Surgery was performed through a posterior approach for an indication of osteoarthritis. Ten weeks later, he felt his left hip dislocate as he got up from a seated position. Imaging at this time demonstrated an anterior prosthetic hip dislocation (Figures 1A, 1B), and it was successfully reduced by the emergency department (ED).

**Figure 2:**
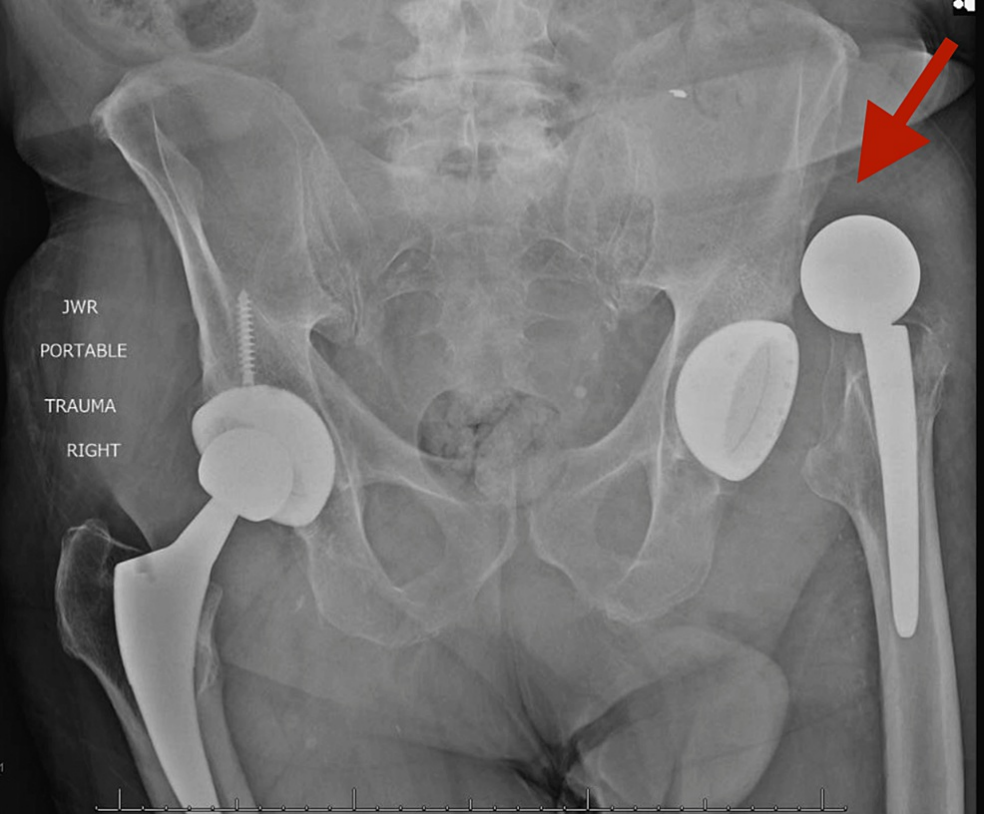
AP pelvis radiograph demonstrating superior dislocation of left THA AP, anterior-posterior; THA, total hip arthroplasty

He sustained a second anterior dislocation, and it was successfully reduced again by the ED 19 days after his second dislocation. His third dislocation occurred just two days after his second, and he was seen and evaluated by our orthopedic team. Imaging demonstrated a superior dislocation of the hip (Figure 2).

**Figure 3:**
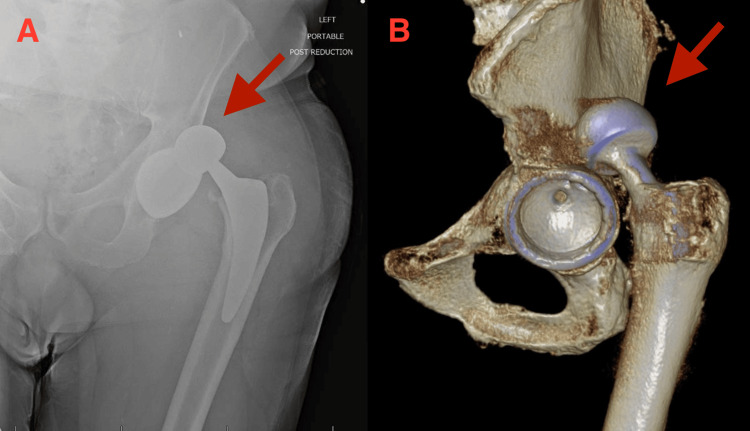
AP left hip radiograph (A) and three-dimensional CT scan view (B) demonstrating superior dislocation of left THA AP, anterior-posterior; THA, total hip arthroplasty; CT, computed tomography

At that time, the patient had no motor or sensory deficits. A closed reduction attempt under conscious sedation in the ED was made; however, even after several tries, it was unsuccessful. The patient then began to exhibit symptoms of sciatic nerve palsy. Radiographs and computed tomography (CT) scan demonstrated a posterior superior dislocation (Figures 3A, 3B).

The patient was taken to the operating room (OR) later that day for another closed reduction attempt, but this was unsuccessful again. Fluoroscopy was used during the closed reduction attempt in the OR, and the hip was found to be very unstable, transitioning from anterior inferior to posterior dislocation positions. Examination following this attempt revealed a sciatic nerve palsy, and the patient was noted to have no function in the deep peroneal, superficial perineal, and tibial divisions. The patient was promptly placed in Buck’s traction to decrease suspected compression of the sciatic nerve by the femoral head, and some patchy sensation to the foot began to return.

The following day, the patient was taken back to the OR for open reduction, possible revision THA, and exploration. A posterior approach was used, incorporating the previous incision. Significant soft tissue damage was noted, including a tear of over 75% of the gluteus medius of the trochanter. In the circumferential periacetabular area, there was profound soft tissue injury. A large portion of the abductor muscle bellies had been stripped off the ilium, and the short external rotators were stripped as well, with soft tissue stripping down to the ischium. A large hematoma was evacuated, and some of the posterior capsule was found to be entrapped in the acetabulum. The sciatic nerve was identified and found to have a complete transection of the tibial segment medially and a partial transection of the fibular component laterally. The lateral portion of the nerve had a 25% disruption of its most medial surface. Plastic surgery then performed epineural repair of the sciatic nerve with neurolysis and wrapped the ends of the nerve with a nerve protector wrap.

**Figure 4:**
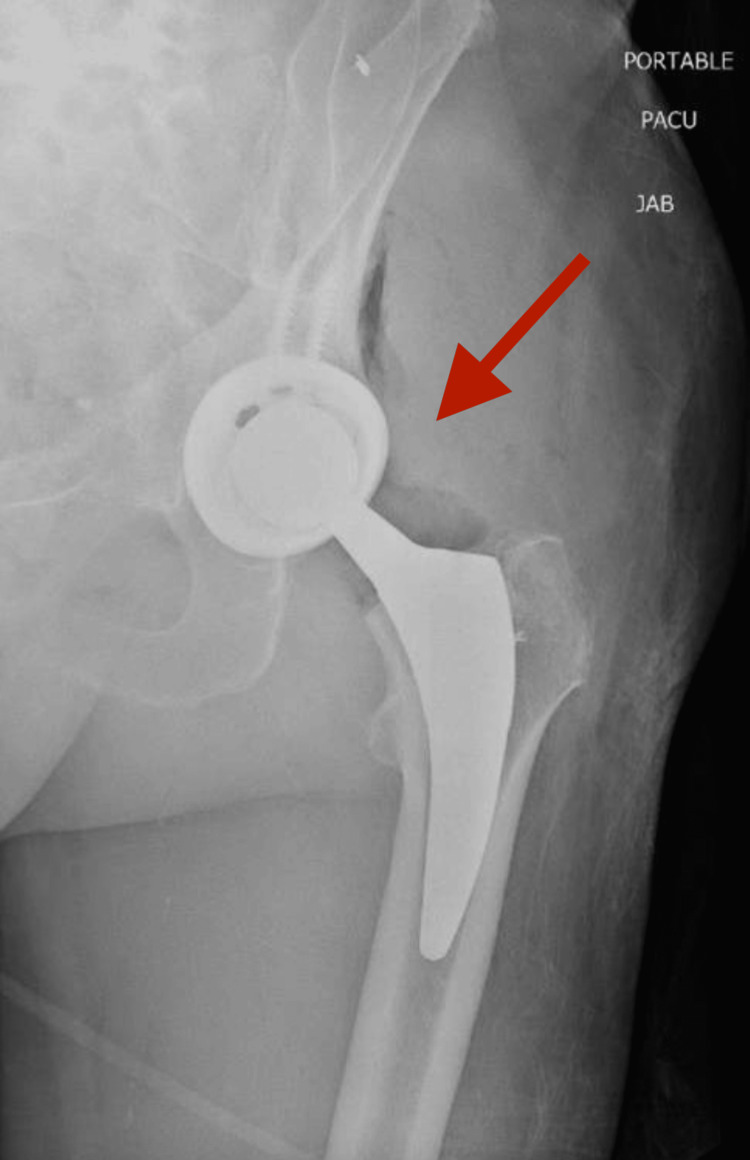
AP radiograph of left hip demonstrating concentric reduction of left THA AP, anterior-posterior; THA, total hip arthroplasty

Stability with the hip reduced was checked thoroughly and was found to be stable with flexion to 90° and internal rotation to 45° with adduction. The hip was also stable when extended and externally rotated; however, there was impingement posteriorly, causing anterior instability. The femoral head was removed, revealing the trunnion to be in good condition. The femoral stem appeared to have an appropriate version and was well-fixed. The acetabular component was also well-fixed, though the inclination of the cup was slightly vertical. Due to the severe soft tissue compromise, it was felt that a constrained liner was going to be necessary to improve stability. For this reason, the acetabular component was not removed to prevent the risk of bone loss and potential rip-out of the new cup (Figure 4).

Postoperatively, the patient was placed in a hip abduction brace as well as an ankle-foot orthosis (AFO). The patient remained without motor function in the lower leg, and sensation was patchy below the knee up to the ankle. There was no return of sensation distal to the ankle. The patient was placed on indomethacin for heterotopic ossification prophylaxis and started on gabapentin. He also utilized a transcutaneous electric nerve stimulation (TENS) unit. At the three-month follow-up, he remained without any signs of nerve recovery and was ambulating with a walker.

## Discussion

Sciatic nerve injuries are rare, but there can be significant complications associated with the closed reduction of a dislocated THA. We present a patient who developed a substantial sciatic nerve laceration following a closed reduction procedure. To our knowledge, there are nine case reports in the literature of sciatic nerve injury after closed reduction of THA [2,7-14]. Of these eight, only Jolissaint et al. describe a case that resulted in a laceration of the sciatic nerve [8]. Their patient sustained a laceration of the sciatic nerve involving approximately 60% of the nerve [8]. Given the low possibility of successful muscle reinnervation prior to muscle death, they opted not to repair the nerve. Of the remaining seven cases, five describe the sciatic nerve wrapping anteriorly around the femoral neck, one describes a traction injury, and Lazansky does not describe a mechanism of nerve injury [2,7-14]. Only one of these cases reports a patient to have recovery of nerve function [2].

There seems to be a higher risk of sciatic nerve injury and entrapment following closed reduction of a dislocated THA in a patient who has undergone revision surgery [8]. There is a known higher rate of dislocation and instability in revision THA. The altering of soft tissues and distortion of normal anatomy following revision surgery may place the nerve at increased risk. While the patient in our case did not have revision surgery prior to dislocation, they did sustain multiple episodes of instability and underwent multiple reduction attempts, likely causing repetitive trauma to both the nerve and the soft tissues. Each of the other cases also describes sciatic nerve injury largely with posterior dislocation [2,7-14]. Jolissaint et al. reported their case to be a posterior dislocation; however, radiographs during successive attempts at unsuccessful reduction showed a very unstable hip with the femoral head dislocated anteriorly, posteriorly, superiorly, and inferiorly at times throughout repetitive reduction maneuvers [8]. The patient in our case first sustained two anterior dislocations that were successfully reduced; however, his third dislocation was posterior superior and was irreducible. During the closed reduction attempt in the OR with fluoroscopy, the hip was grossly unstable, and the femoral component was noted to translate from anterior inferior to posterior superior. While initially, the patient may have had anterior instability, but he developed posterior instability likely because of the soft tissue damage sustained from repeated dislocations and reduction maneuver.

Closed reduction is a commonly performed procedure to treat dislocated THA, with the aim of restoring joint stability and function. While this process is not benign and the sciatic nerve can be susceptible to injury during reduction, there is also a higher risk of major sciatic nerve injury in patients with prolonged hip dislocation. Many maneuvers for closed reduction of hip dislocations exist and are utilized under different circumstances, as each case may present its own unique challenges. It is important to proceed with caution during closed reductions of THAs and to use clinical judgment if multiple attempts are continuously unsuccessful. Forceful or repeated attempts may lead to hematoma or damage to the surrounding soft tissue structures, which may displace the nerve anteriorly, placing it at greater risk of entrapment and injury [14]. Six anatomic variants have been described relating to the sciatic nerve and its course in the piriformis [1]. Some of these variants may also displace the nerve anteriorly closer to the hip joint and result in a higher risk of injury during closed reduction maneuvers. As Jolissaint et al. discussed, a forced reduction maneuver can likely cause a direct laceration or avulsion to the sciatic nerve, depending on its positioning at the time [8]. The sciatic nerve may become more at risk if it becomes entrapped between the acetabular cup and the femoral head or compressed against the edge of the cup during a reduction maneuver.

Diagnosing sciatic nerve injury following closed reduction can be challenging, as symptoms may be nonspecific or overshadowed by pain related to the underlying hip pathology. Common clinical manifestations include lower limb weakness, sensory deficits, and neuropathic pain. If the patient has severe pain and complete motor palsy, this may be more suggestive of nerve entrapment, and we agree with advocating for early surgical exploration, as discussed by Chan et al. [7]. In this case, the patient presented with immediate post-reduction neurological deficits, including foot drop and sensory loss along the posterior aspect of the lower leg and foot. Early recognition of sciatic nerve injury is crucial to optimize treatment outcomes. A thorough neurological examination, including motor and sensory assessments, should be performed both pre- and post-reduction. A change in neurologic examination directly following a reduction attempt may prompt surgical exploration on a more expedited timeline. If the diagnosis remains unclear, magnetic resonance imaging (MRI) may be helpful in visualizing nerve edema, hematoma, direct nerve injury, or entrapment. Cheung et al. describe the role of metal-suppression MRI in detecting sciatic nerve entrapment around the femoral stem following closed reduction of a THA [15]. Metal-suppression MRI allows for better evaluation of the bone-metal interface as well as soft tissue pathology [15]. CT may be useful for the evaluation of any possible hematoma, as nerve compression by a hematoma is on the differential and may indicate a necessity for early surgical exploration [2].

Management of sciatic nerve injury following closed reduction should be individualized based on the severity and location of the lesion. One study reported that nearly 75% of patients developed partial recovery with good functional outcomes when the sciatic nerve was found to be intact; however, over 25% of this population had no nerve recovery [16]. Unfortunately, there is little in the literature about long-term outcomes or management of complete sciatic nerve lacerations. Maripuu et al. described partial nerve recovery in a patient following sciatic nerve laceration treated with neurolysis and autograft [17]. In the setting of sharp lacerations of peripheral nerves without large defects, direct tensionless end-to-end repair results in the most predictable outcomes [18]. A large peripheral nerve such as the sciatic nerve typically heals at a rate of 2-5 mm per day; therefore, recovery could take several months, and full motor recovery is unlikely following a lack of motor innervation over six months [19]. Conservative management, including physiotherapy, analgesia, and close monitoring, may be appropriate for milder injuries, such as neuropraxia. More severe injuries, such as nerve transection or significant stretch injuries, may require surgical intervention, functional bracing, and possibly tendon transfers in the future if needed [20]. If pursuing nerve repair, this should be done within two to three days following injury to decrease fibrosis, limit retraction, and help facilitate regrowth [18].

In our case, the patient underwent urgent surgical exploration, revealing a near-complete laceration of the sciatic nerve. Primary nerve repair was performed using microsurgical techniques, and the patient was subsequently started on a rehabilitation program with functional bracing. The patient did not demonstrate any evidence of nerve recovery at the three-month follow-up.

## Conclusions

In summary, sciatic nerve injury following closed reduction of a dislocated THA is a rare but potentially debilitating complication. Healthcare professionals in the orthopedic surgery and ED fields should be aware of the risk factors, potential mechanisms of injury, and diagnostic challenges associated with this complication. Early recognition, accurate diagnosis, and prompt management, including surgical intervention when indicated, are essential for optimizing patient outcomes and facilitating any possible nerve recovery. These injuries fall on a spectrum from minor neuropraxias to near-complete transections, and we aim to educate our readers on the true severity that these injuries can reach. Further studies and larger case series are warranted to better understand the incidence, risk factors, and optimal management strategies for this uncommon complication.
